# Wildlife target detection based on improved YOLOX-s network

**DOI:** 10.1038/s41598-024-73631-1

**Published:** 2024-10-09

**Authors:** Bao XiaoAn, Zhou LinQing, Tu XiaoMei, Wu Biao, Zhang QingQi, Jin YuTing, Zhang Na

**Affiliations:** 1 School of Computer Science and Technology, Zhejiang Sci-Tech University, Hangzhou, 310018 Zhejiang China; 2 School of Information Science and Engineering, Zhejiang Sci-Tech University, Hangzhou, 310018 Zhejiang China; 3 School of Science, Zhejiang Sci-Tech University, Hangzhou, 310018 Zhejiang China; 4 School of Civil Engineering and Architecture, Zhejiang Guangsha Vocational and Technical University of construction, Dongyang, 322100 Zhejiang China; 5 The Graduate School of East Asian Studies, Yamaguchi University, Yoshida 1677-1, Yamaguchi-shi, Yamaguchi-ken 753-8540 Japan

**Keywords:** Wildlife detection, MobileViT-Pooling, YOLOX-s, Dynamic head, Focal-IoU loss function, Computational biology and bioinformatics, Mathematics and computing

## Abstract

To addresse the problem of poor detection accuracy or even false detection of wildlife caused by rainy environment at night. In this paper, a wildlife target detection algorithm based on improved YOLOX-s network is proposed. Our algorithm comprises the MobileViT-Pooling module, the Dynamic Head module, and the Focal-IoU module.First, the MobileViT-Pooling module is introduced.It is based on the MobileViT attention mechanism, which uses a spatial pooling operator with no parameters as a token mixer module to reduce the number of network parameters. This module performs feature extraction on three feature layers of the backbone network output respectively, senses the global information and strengthens the weight of the effective information. Second, the Dynamic Head module is used on the downstream task of network detection, which fuses the information of scale sensing, spatial sensing, and task sensing and improves the representation ability of the target detection head. Lastly, the Focal idea is utilized to improve the IoU loss function, which balances the learning of high and low quality IoU for the network. Experimental results reveal that our algorithm achieves a notable performance boost with mAP@0.5 reaching 87.8% (an improvement of 7.9%) and mAP@0.5:0.95 reaching 62.0% (an improvement of 5.3%). This advancement significantly augments the night-time wildlife detection accuracy under rainy conditions, concurrently diminishing false detections in such challenging environments.

## Introduction

As a valuable resource provided by nature, wildlife not only possesses commercial value but also holds important ecological and scientific significance. Wildlife resources are widely distributed across China. The identification and detection of wildlife facilitate an understanding of the habits, habitats, and population distribution of wildlife. This not only enriches our knowledge of wildlife but also supports the special protection of China’s endangered species^1^. The emergence of infrared cameras has been a major advancement in wildlife investigation methods. Over the past few years, infrared cameras have enabled researchers to record real scenes of many wildlife activities in the field^2^. In recent years, with the development of artificial intelligence, computer vision has increasingly entered the public’s field of view. Widely used in transportation, health care, agriculture, and other fields^3,4^, computer vision enables researchers to better protect and study wildlife.

There are few deep learning target detection algorithms specifically applied to wildlife detection^5^. Peter^6^ used YOLO Object Detection to identify hares and roe deer in thermal aerial video footage. Mina Gabriel^7^ and colleagues employed YOLOv3 for detecting cats, birds, rats, and lizards, yet this method suffered from a narrow range of recognition categories and lower accuracy.

Roy^8^ proposed an automated high-performance detection model, WilDect - YOLO, based on deep learning for real-time detection of endangered wildlife. In this model, a residual block is introduced into the CSPDarknet53 backbone network to extract strong and distinctive deep spatial features, and DenseNet blocks are integrated to improve the preservation of critical feature information.Schneider applied the Faster R-CNN target detection algorithm on the Reconyx Camera Trap^9^ and Snapshot Serengeti^10^ datasets, achieving high detection accuracy but at a slower speed. Zhenan Cheng^11^ introduced a self-attentive deep residual network and improved the data equalization loss function of Faster R-CNN for a wildlife dataset he established in the Inner Mongolia Sai Reserve, which includes species like lynx, wild boar, wildebeest, zebra antelope, horse deer, mink, and roe deer. To address the challenges of low detection rates in nighttime scenes, He Jia^12^ proposed a mechanism of infrared image domain migration using the generative adversarial network model, which enhanced the detection accuracy in nighttime infrared scenarios. Addressing the long-tail problem in wildlife datasets, Cai Qianzhou^13^ developed a network model based on YOLOv4-tiny, combining transfer learning and reweighting to improve accuracy with limited sample images, although the overall detection accuracy remains suboptimal. Zhang^14^ proposed an enhanced animal detection algorithm based on YOLOv5 to address the issues of low detection accuracy and slow detection speed when automatically detecting and classifying large animals in natural environments.

Target detection algorithms based on deep learning are categorized into two types: one-stage algorithms and two-stage algorithms^15^. One-stage algorithms directly compute and predict both the classification and location information of the targets, offering high real-time performance. Typical examples include SSD (Single Shot Multibox Detector)^16^ and YOLO (You Only Look Once)^17^. In contrast, two-stage detection algorithms first generate candidate regions containing potential objects. These regions are then classified and verified using a convolutional neural network model to produce the final predictions. Representative algorithms include Region-based Convolutional Neural Networks (R-CNN)^18^, Spatial Pyramid Pooling Networks (SPP-NET)^19^, Fast R-CNN^20^, and Faster R-CNN^21^. The YOLO algorithm, a notable one-stage method, utilizes a convolutional kernel on the feature map to predict the class and coordinates of bounding boxes at various scales, incorporating different pre-set anchor box sizes to enhance detection accuracy. YOLO is known for its compact model size and high speed, making it suitable for mobile applications^22^. In comparison, two-stage algorithms generally achieve greater accuracy in both detection and localization, while one-stage algorithms excel in processing speed.

**Fig. 1:**
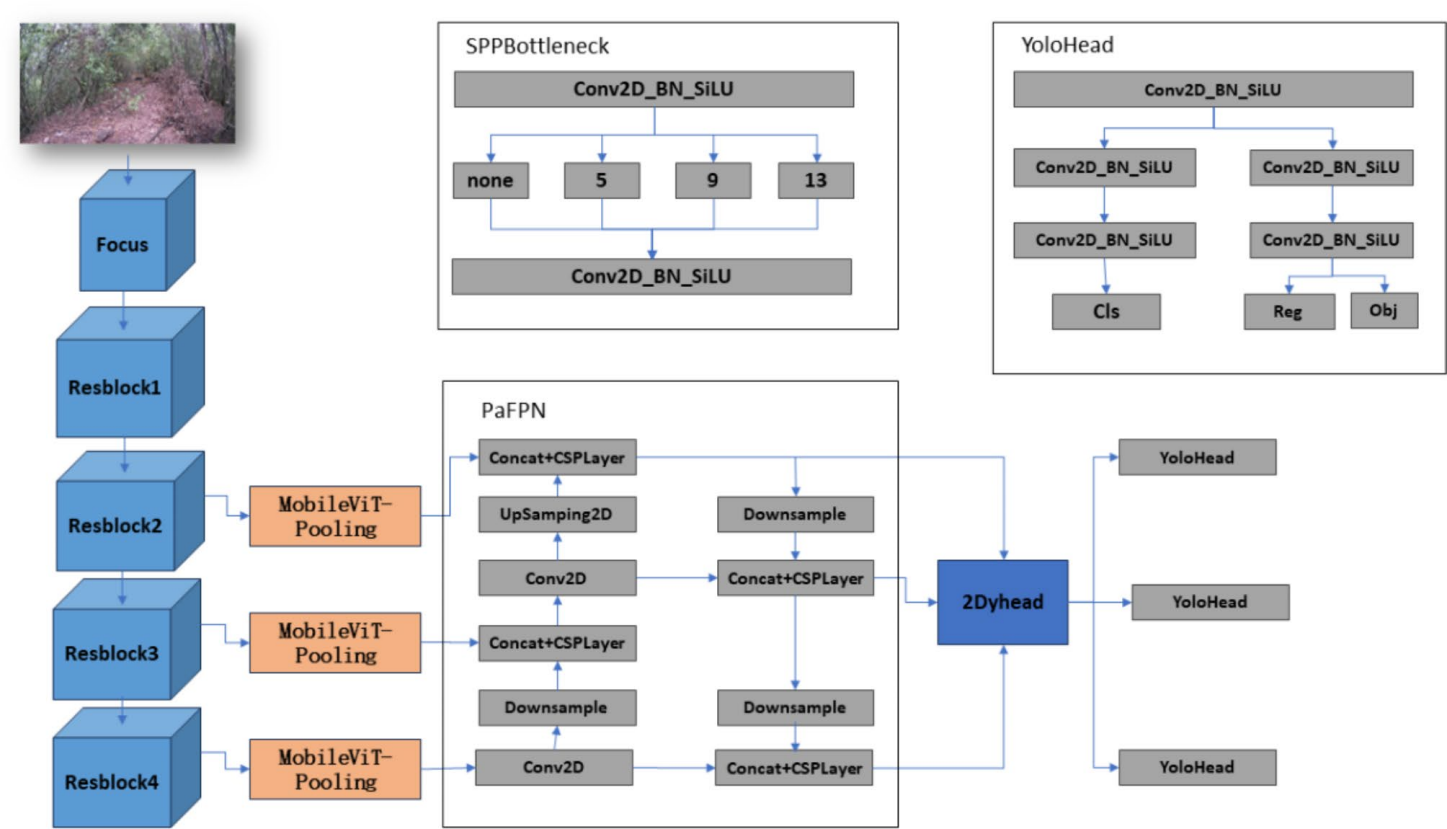
This is the improved YOLOX-s network structure diagram.

In the practice of wildlife detection, the conventional single-stage YOLOX-s network, while fast and efficient, still needs improvement in accuracy under complex conditions such as nighttime or rainy weather. To enhance the detection performance of the YOLOX-s network in challenging environments, this study introduces three key technological innovations aimed at optimizing network performance and reducing false detections. Firstly, the MobileViT-Pooling architecture enhances the multi-head self-attention module in MobileViT by incorporating pooling operations. This hybrid method of attention mechanisms and pooling not only optimizes the feature extraction process but also significantly reduces the model’s parameter count without sacrificing accuracy. This technique is particularly suited for in-depth analysis in environments with rich and variable features, such as during nighttime or rainy conditions for wildlife monitoring. Secondly, the dynamic detection head (DyHead) integrates feature layers that are scale-aware and spatially aware, along with task-aware output channels. By applying different attention mechanisms at these various levels, DyHead complements performance across scales, significantly enhancing the representational capability of the detection head. This strategy of dynamically adjusting attention allows the network to better adapt to targets of varying sizes, enhancing detection precision and efficiency. Lastly, in response to the issue of loss fluctuations caused by low-quality samples in traditional IoU Loss, this study adopts an IoU Loss function improved with the Focal concept. By adjusting the penalty weights for high-quality and low-quality samples, the modified loss function stabilizes model optimization and reduces false detections, especially in visually challenging conditions. The structure of the improved algorithm presented in this paper is shown in Fig. 1.

## Improved YOLOX-s

### YOLOX

The YOLOX series represents single-stage target detection algorithms known for their fast inference and real-time performance. YOLOX utilizes an Anchor-Free design concept, predicting the location and category of targets directly through the network, which reduces the limitations associated with anchor frames and enhances detection performance. Ge et al.^23^ introduced the YOLOX algorithms, which include YOLOX-s, YOLOX-m, YOLOX-l, and YOLOX-x variants. The architecture of YOLOX is divided into three main components: Backbone, Neck, and Head. The Backbone is built on the CSPDarknet^24^ network, where the input image undergoes initial feature extraction. This layer yields three feature layers essential for subsequent network construction. The Neck, consisting of the PAFPN (Path Aggregation Network with Feature Pyramid Network), acts as YOLOX’s Enhanced Feature Extraction Network. It fuses the three effective feature layers from the Backbone, aiming to amalgamate feature information across different scales. In the PAFPN, these layers are further utilized for continued feature extraction. The Head segment incorporates a Decoupled Head, which offers enhanced expressive capability and faster network convergence.

### MobileViT-pooling

**Fig. 2:**
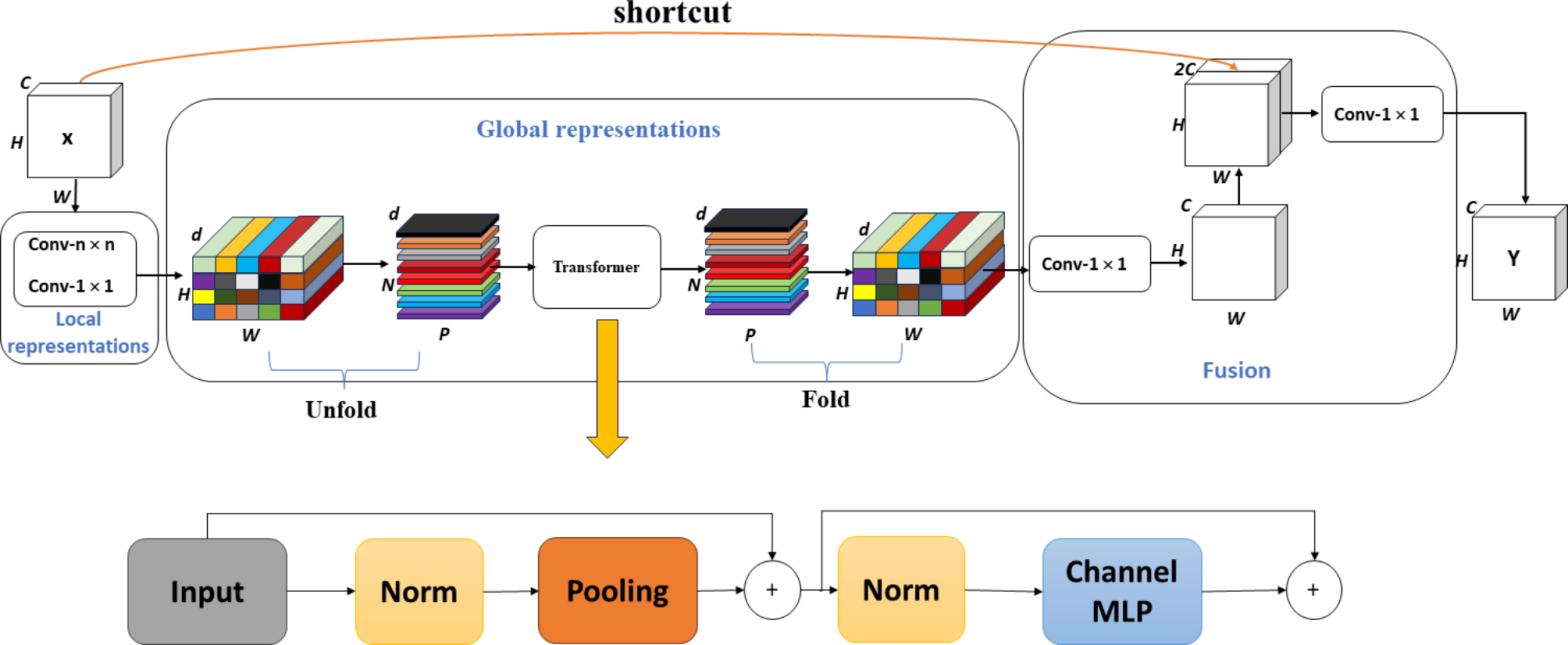
MobileViT-Pooling module structure diagram.

MobileViT^25^ is a lightweight, general-purpose visualization Transformer designed for mobile devices that utilizes a multi-layer Transformer encoder to process sequences in chunks. Nevertheless, employing multiple Transformer layers can increase the model’s parameter count and slow down detection speeds in practical applications. Excessive layers may also distort the extracted features. In this paper, the improved MobileViT-Pooling (referenced in Fig. 2) employs just a single Transformer layer. The authors of MetaFormer^26^ attribute the success of both the Transformer and MLP models to the generalized architecture of MetaFormer. To further reduce the parameter count and computational burden of the Transformer, we have transformed the multi-head self-attention structure of the Transformer into a non-parameterized spatial pooling operator, which serves as the token mixer module. This modification does not significantly impact detection accuracy in our experiments. Importantly, we recognize that MobileViT’s feature extraction capability is essential for detecting wildlife in this study. Yet, incorporating MobileViT substantially increases the computational demands of the network. Adhering to the principles of being quick, popular, and cost-effective, we have replaced the multi-head self-attention modules in MobileViT with global pooling operations, effectively reducing the network’s computational demands without overly compromising detection accuracy.

In the described model, the feature map initially passes through a convolutional layer with a convolutional kernel size of n × n. This is followed by a channel adjustment using a 1 × 1 convolutional kernel. Subsequently, global feature modeling is performed using the Unfold, Transformer, and Fold structures consecutively. The channel is then adjusted back to its original size using a convolutional layer with a 1 × 1 kernel size. This adjustment is succeeded by shortcut branching and channel-wise concatenation of the original input feature maps, concluding with a feature fusion through a convolutional layer with a kernel size of *n* × 1 to produce the final output.

In this paper, we explore the potential of merging the strengths of CNNs and ViTs. CNNs are proficient in handling localized image features and structural information, while ViTs excel in managing long-range dependencies and global contextual information. Combining these two technologies may lead to superior performance. For this study, we designed two distinct models integrating CNN and ViT technologies:

#### Algorithm 1

Incorporates ViT within the CNN structure. In this hybrid model, CNN and ViT modules are alternated within the network architecture. This configuration may necessitate additional techniques and experiments to optimize the integration of CNN and ViT.

#### Algorithm 2

Employs ViT after CNN processing. Initially, features are extracted using the CNN, and then these features are fed into the ViT for more complex processing and inference. This arrangement leverages the ViT’s capability to handle global contexts and long-range dependencies, while retaining the CNN’s advantages for processing local features. The outcomes of this approach are detailed in Table 1.

**Table 1:** Experimental comparison results of Algorithm 1 and algorithm 2.

method	Params/M	FLOPs/G	mAP@0.5/%	mAP@0.5:0.95/%
Algorithm 1	22.4	51.2	76.3	54.6
Algorithm 2	22.4	51.2	**83.2**	**57.6**

Based on the experimental results, the approach of using ViT after CNN significantly outperforms embedding ViT within the CNN architecture. The difference in mAP@0.5 is 6.9%, and the gap in mAP@0.5:0.95 is 3.0%. We believe that employing ViT after processing with CNN allows the model to better segregate and differentiate various types of information. The CNN focuses on processing low-level and mid-level features, enabling ViT to concentrate on handling high-level and abstract features subsequently. This sequential processing might enhance the model’s performance and generalization capabilities. In contrast, embedding ViT directly within the CNN complicates the integration, as the alternating modules may not optimally handle the distinct features of the dataset. Specifically, in datasets where the features of wild animals often resemble environmental backgrounds, multiple embeddings of the ViT module within the CNN could lead to interactions of global information that might distort the already extracted features.

### Dynamic head

**Fig. 3:**
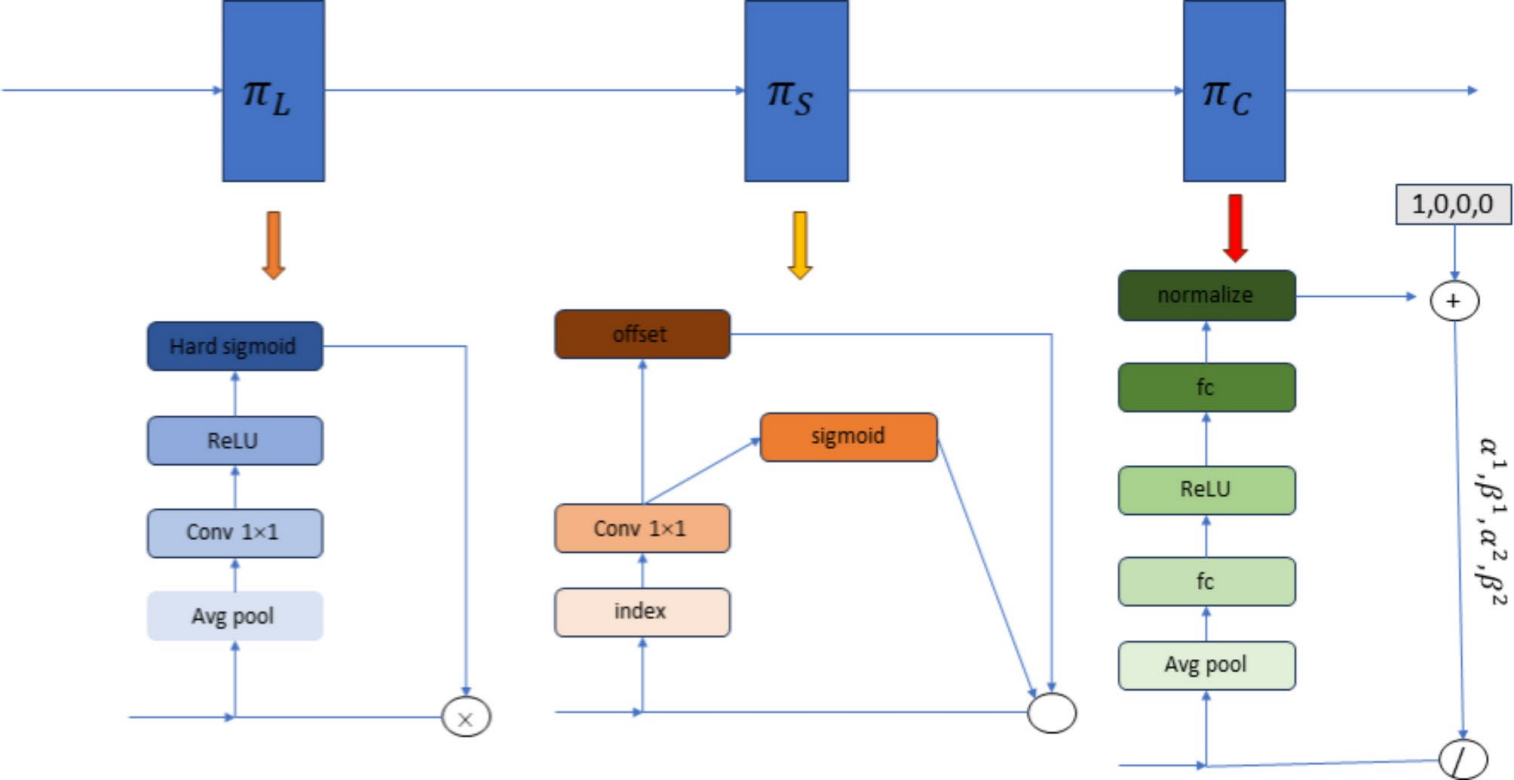
Dynamic head structure diagram.

Environmental background transformations pose significant challenges in visual target detection and tracking. Dynamic weather conditions, such as rainy nights, often introduce substantial noise into images, complicating the task of accurately recognizing wildlife. To address this, our paper introduces the ‘Dynamic Head‘^27^—a network structure designed to manage dynamic changes and focus attention effectively. The structure of the Dynamic Head is illustrated in Fig. 3. It primarily leverages an attention mechanism to enhance the model’s three key perceptual capabilities, thus improving detector performance. The structure of the DyHead block is shown in the following figure, where $\:{\pi\:}_{L}$, $\:{\pi\:}_{S}$, and $\:{\pi\:}_{C}$ represent scale-aware attention (Level-wise), spatial-aware attention (Spatial-wise), and task-aware attention (Channel-wise), respectively.

The introduction of the Dynamic Head brings several advantages to the research discussed in this paper. Nonetheless, determining the optimal number of layers to stack in the Dynamic Head, as well as understanding the specific effects of different layer counts on model performance, requires consideration of the specific model, task, and dataset involved. Increasing the number of layers can enhance the model’s complexity, potentially boosting performance but also increasing the risk of overfitting. Conversely, reducing the number of layers may simplify the model and reduce overfitting, but could compromise performance. To address this, we experimented with various stacking configurations, aligning our changes with the original author’s design. The results of these experiments are documented in Table 2.

**Table 2:** Experimental results of different dyhead stacking times.

Number of stacks	Params/M	FLOPs/G	mAP@0.5	mAP@0.5:0.95
1	9.4	28.8	80.42	56.82
2	9.4	29.9	**81.58**	**58.36**
4	9.4	32.1	78.23	54.76
6	9.4	34.4	76.18	53.45

At a stacking number of 2, mAP@0.5 and AP@0.5:0.95 reaches a maximum of 58.36 and 81.58, respectively. this indicates that stacking 2 layers of DyHead performs best on this wildlife detection task. When the number of stacks exceeded 2, the mAP of the model started to decrease, which could be due to too many stacks causing the model to overfit or making the optimization of the model more difficult. Therefore, for this wildlife detection task, stacking 2 layers of DyHead may have been the best choice, as it achieved the highest mAP while keeping the number of parameters constant, albeit with a slight increase in computation.

### Focal-IoU loss

IoU Loss: Intersection over Union (IoU) is a commonly used metric for evaluating target detection results, which measures the degree of overlap between the predicted bounding box and the actual bounding box. During the training process, the predicted bounding box can be made closer to the real bounding box by minimizing the IoU Loss.The IoU-based function is as follows:

1 $$\:\begin{array}{c}L\left(B,{B}^{gt}\right)=1-\frac{\left|B\cap\:{B}^{gt}\right|}{\left|B\cup\:{B}^{gt}\right|}+R\left(B,{B}^{gt}\right)\end{array}$$

where B denotes the detection frame and $\:{B}^{gt}$ denotes the true box, *the*$\:R\left(B,{B}^{gt}\right)$ penalized items.

Focal Loss^28^ is primarily employed to address the issue of category imbalance in classification tasks, which involves discrepancies in the number of positive (target) and negative (non-target) samples. In target detection tasks, the number of negative samples often far exceeds that of positive samples. Focal Loss works by reducing the weight of easily classified samples (mostly negative samples) and increasing the weight of challenging-to-classify samples (such as occluded targets or targets resembling the background). This adjustment enables the model to prioritize attention to these challenging samples during the training process. The formula is represented as follows:

2 $$\:\begin{array}{c}FocalLoss=-{\alpha\:}_{t}{\left(1-{p}_{t}\right)}^{\gamma\:}\text{log}\left({p}_{t}\right)\end{array}$$

where $\:{\alpha\:}_{t}$ is used to solve the problem of positive and negative sample imbalance,γ is a parameter in the range [0,5], *and*$\:{\left(1-{p}_{t}\right)}^{\gamma\:}$ is used to solve the problem of unbalanced hard and easy samples.

An ideal loss function should have these characteristics: Small regression errors should yield low gradients, indicating minimal parameter adjustments. Conversely, large errors should produce high gradients, necessitating significant parameter changes. The algorithm often generates a large number of ineffective prediction boxes during prediction. In order to enhance the weight of high-quality prediction boxes in the IoU loss function and improve the quality of training, we have applied the Focal concept to IoU, resulting in the proposal of the Focal-IoU function. Furthermore, the proposed Focal-IoU loss function integrates the concept of Focal, aiming to fine-tune the error rates in cases of small errors, while making drastic adjustments in cases of large errors:

3 $$\:\begin{array}{c}{L}_{Focal-IoU}={IoU}^{\gamma\:}{L}_{IoU}\end{array}$$

**Fig. 4:**
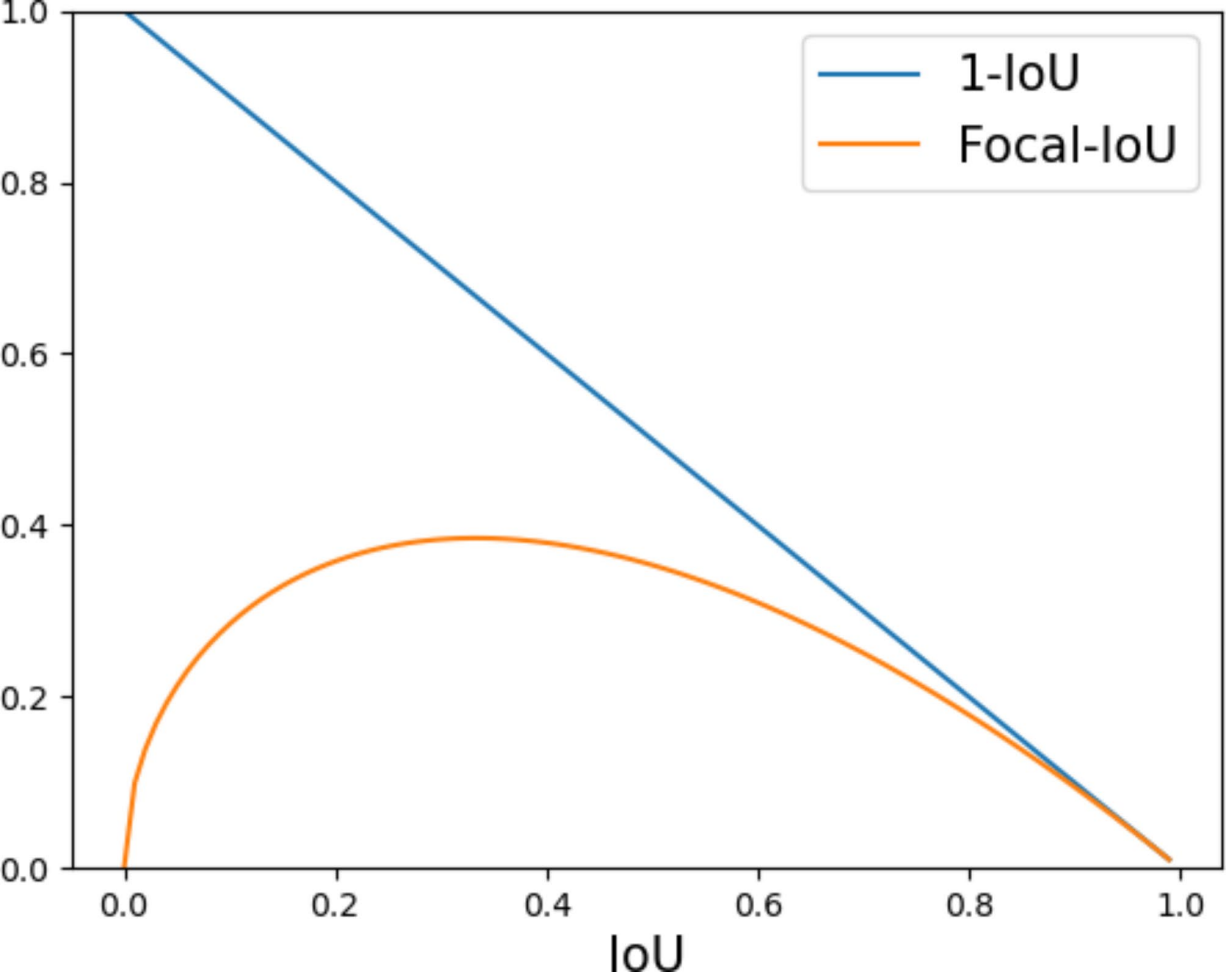
1-IoU and Focal-IoU curves.

γ is a hyperparameter, which we set to 0.5 for the $\:\left(1-IoU\right)$ curve. The orange color represents $\:Io{U}^{0.5}\left(1-IoU\right)$. For IoU values in the range of 0 to 0.8, $\:Io{U}^{0.5}\left(1-IoU\right)$ decreases, while for IoU values in the range of 0.8 to 1, the color of $\:Io{U}^{0.5}\left(1-IoU\right)$ remains essentially unchanged. Figure 4 illustrates that the Focal-IoU loss enables the network to focus more on simple samples by reducing the loss associated with difficult samples.

The design objective of the Focal-IoU Loss is to tackle both the IoU data imbalance issue and enhance the localization accuracy of the prediction frame simultaneously. To evaluate the impact of the Focal-IoU Loss, we utilize this dataset and assess whether it leads to improved performance under identical conditions.

**Table 3:** Experimental results for different IoU loss functions.

IoU loss function	mAP@0.5/%	mAP@0.5:0.95/%
IoU	79.9	**56.7**
Focal-IoU	**80.5**	56.4
GIoU^29^	78.5	56.2
DIoU^30^	77.9	54.9
CIoU^30^	75.6	53.5

In Table 3, it is evident that the model employing Focal-IoU Loss achieves the highest performance on the mAP@0.5 metric, reaching 80.5%. However, for the mAP@0.5:0.95 metric, the performance of Focal-IoU Loss is comparable to that of IoU Loss but slightly lower. This suggests a slight decrease in the detection accuracy of targets with Focal-IoU Loss. The inferior detection accuracies of several other IoU loss functions, such as GIoU, DIoU, and CIoU, imply that they are less suited to this problem compared to Focal-IoU Loss and IoU Loss. Overall, Focal-IoU Loss demonstrates relatively strong performance in this wildlife target detection problem, particularly at larger IoU thresholds.

## Experimental results and analysis

### Experimental environment

The computer in the experiment was configured as follows: the CPU was an Intel(R) Xeon(R) Gold 5218R CPU at 2.10 GHz, the RAM was 125 GB, the system was Linux 4.15.0-142-generic x86_64-bit, and the GPU was an NVIDIA Corporation Device 2204 [ GeForce RTX 3090]. python version 3.8.12, cuda version 11.0, pytorch version 1.7.1.

The experiments in this section employed the YOLOX-S network, which utilizes a pre-trained model from COCO. The inserted MobileViT-Pooling module was trained from scratch. The network parameters were configured as follows: a momentum parameter of 0.9, an initial learning rate of 0.01 with a minimum value of 0.0001, cosine annealing decay of the learning rate during network training, and training for 300 epochs. The first 285 epochs were dedicated to preventing overfitting using data augmentation strategies, and the Adam optimization algorithm was utilized to update the network weights.

### Dataset

**Fig. 5:**
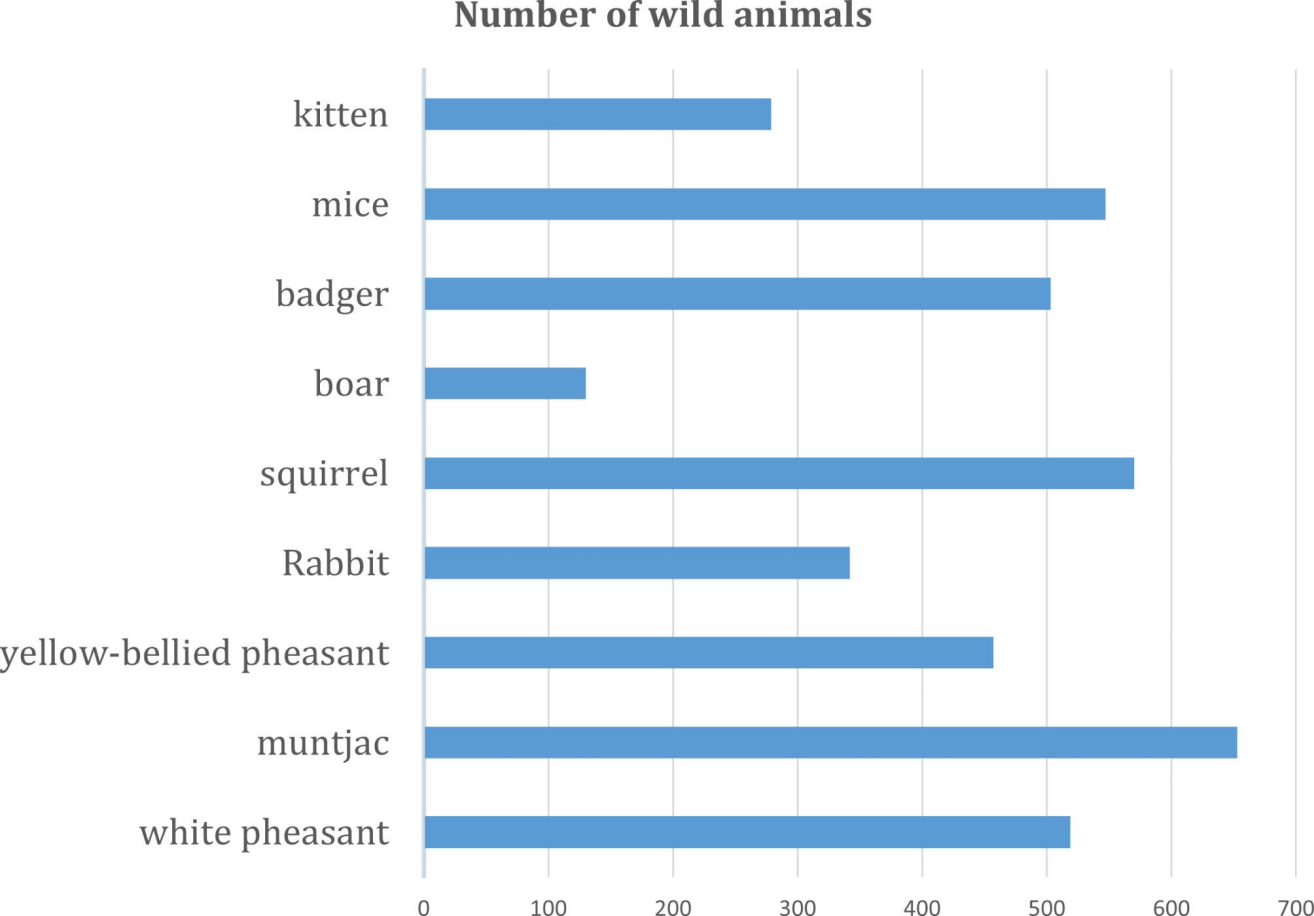
Statistics of wildlife datasets.

Our dataset covers nine different animal species in the reserve, which includes a wide range of animal species including white pheasants, muntjacs, yellow-bellied pheasants and hares. The exact number is shown in Fig. 5.

So far, we have amassed approximately 4000 images of these animals, all captured within their natural habitats, ensuring a high degree of authenticity and biodiversity. Each image captures a genuine moment in nature, documenting the animals’ behavior, ecology, and interactions with other organisms. These image data serve a dual purpose: not only do they facilitate the training and testing of our model, thereby enhancing species recognition accuracy, but they also hold potential for broader biological research endeavors. Such research may include analyzing animal activity patterns, group structures, and more. We partitioned the 4000 images into three sets—training, validation, and testing—using uniform random sampling without intersection. The distribution ratio among these sets is 8:1:1. Our experimental results are based on the mAP@0.5 and mAP@0.5:0.95 metrics evaluated on the test set.

### Ablation experiments and comparison with other SOTA algorithms

During the experimental process, the improved YOLOX-s algorithm was compared with other state-of-the-art (SOTA) algorithms on the wildlife dataset. The mAP@0.5 and mAP@0.5:0.95 were used as evaluation metrics. The experimental results are shown in the Table 4.

**Table 4:** Experimental results of different SOTA algorithms.

Target detection algorithms	image size	Params/M	FLOPs/G	mAP@0.5	mAP@0.5:0.95
YOLOX-s(base)	640 × 640	9.0	26.8	79.9	56.7
+ Focal-IoU Loss (ours)	640 × 640	9.0	26.8	80.5	56.4
+ Focal-IoU Loss + MobileViT-Pooling (ours)	640 × 640	22.4	51.2	85.4	60.2
+ Focal-IoU Loss + MobileViT-Pooling + 2× DyHead(ours)	640 × 640	23.0	56.4	87.8	62.0
YOLOX-m	640 × 640	25.3	73.8	81.9	58.5
YOLOX-l	640 × 640	54.2	155.6	82.4	59.2
YOLOv5-m	640 × 640	21.2	49.0	84.3	60.3
RetinaNet + ResNet50^28^	1280 × 800	36.3	207.9	84.8	60.7
DETR^31^	1280 × 800	41.3	86.0	77.4	52.6
Faster RCNN + ResNet50^21^	1280 × 800	41.2	206.7	86.2	61.4

Starting from the basic YOLOX-s model, we progressively incorporated Focal-IoU loss, MobileViT-Pooling, and DyHead. These additions led to an increase in both the number of parameters and computational complexity. However, they resulted in significant improvements in mAP@0.5 and mAP@0.5:0.95. Notably, the performance notably improved, especially after integrating Focal-IoU Loss and MobileViT-Pooling, indicating the effectiveness of these two techniques for this task. The performance peaked when DyHead was stacked twice.

YOLOX-m and YOLOX-l models, while featuring considerably higher parameters and computational demands compared to YOLOX-s, did not exhibit as pronounced performance enhancements. This suggests some degree of overfitting for these two models in this task.

On the other hand, models such as YOLOv5-m, RetinaNet + ResNet50, DETR, and Faster RCNN + ResNet50 boast larger numbers of parameters and computational complexity. However, their performance does not significantly surpass that of the enhanced YOLOX-s model. This indicates that the YOLOX series offers a better balance between performance and efficiency.

The experimental results demonstrate that the improved YOLOX-s network achieved an mAP@0.5 of 87.8%, marking a 7.9% improvement over the original YOLOX-s. The mAP@0.5:0.95 reached 62.0%, indicating a 5.3% increase.

### Comparative experiment on nighttime datasets

We created a new dataset with only the nighttime images from the original collection. By concentrating on the mean Average Precision (mAP@0.5) metric, we assessed how different algorithms performed in detecting wildlife at night. The data from Table 5 indicate that ours algorithm achieved the highest score of 80.2% in mAP@0.5 among all compared algorithms. This demonstrates that ‘ours’ outperforms in accuracy, precisely locating and identifying animals within the images. Drawing from the mAP@0.5 outcomes, Ours algorithm is determined to be more accurate in nocturnal wildlife detection. This is due to its use of MobileViT-Pooling for feature extraction and the innovative Focal-IoU loss for improved target positioning, as introduced in our study.

**Table 5:** Experimental results of Nighttime datasets.

Target detection algorithms	image size	Params/M	FLOPs/G	mAP@0.5
**Ours**	640 × 640	23.0	56.4	**80.2**
YOLOX-m	640 × 640	25.3	73.8	71.3
YOLOX-l	640 × 640	54.2	155.6	72.4
YOLOv5-m	640 × 640	21.2	49.0	73.4
RetinaNet + ResNet50	1280 × 800	36.3	207.9	71.8
DETR	1280 × 800	41.3	86.0	68.5
Faster RCNN + ResNet50^21^	1280 × 800	41.2	206.7	74.6

### Visual analysis

**Fig. 6:**
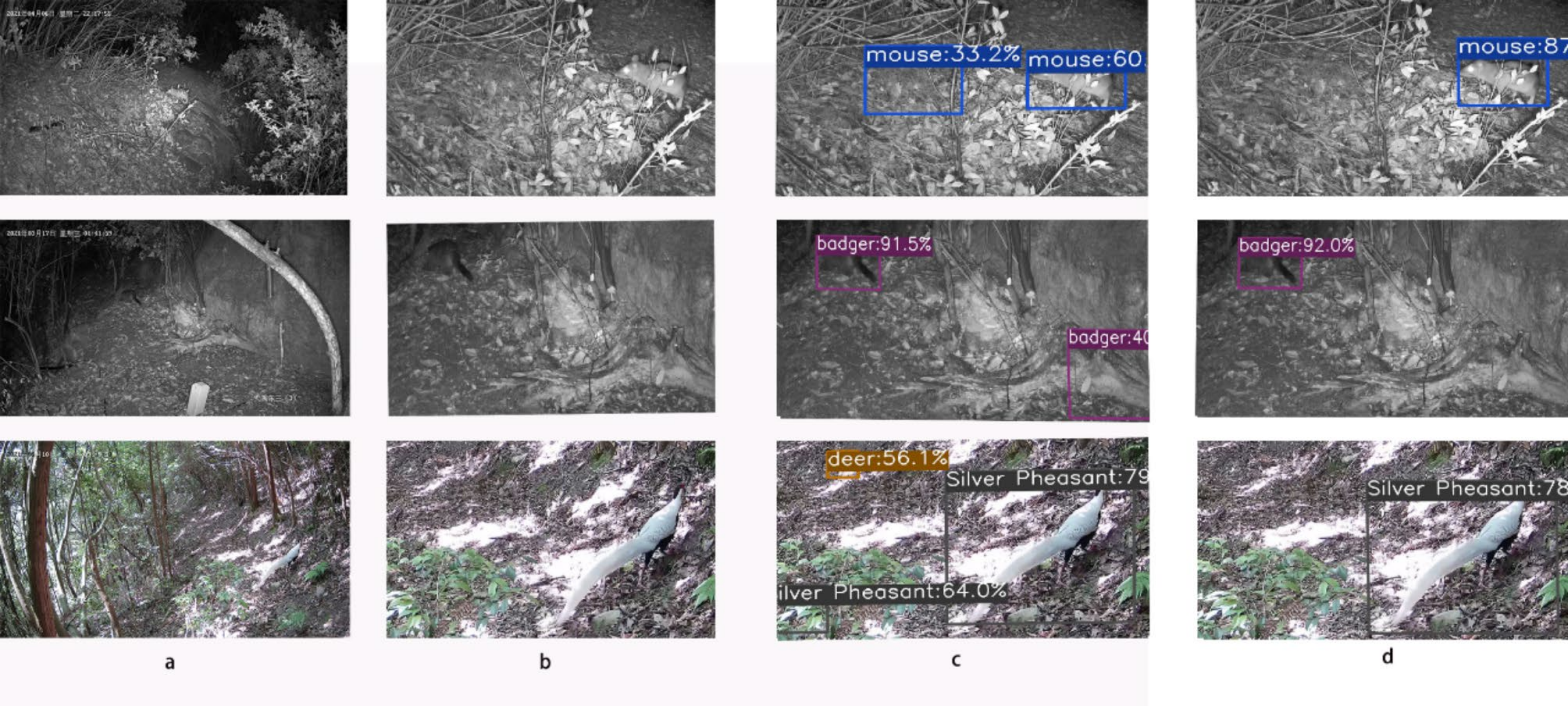
Detection effects presented by different algorithms. (**a**) Original Image. (**b**) Local Image. (**c**) YOLOX-s Detection Result Image. (**d**) Improved YOLOX-s Detection Result Image.

By adding the improved mechanisms such as MobileViT-Pooling, DyHead, and improved Focal-IoU Loss in YOLOX network, the effect of wildlife detection has been improved, and the detection effect of the test set is shown in Fig. 6.

In the first picture, YOLOX-s recognized the raised earth as a mouse in the dark, the fallen tree stump as a badger in the second picture, and the pile of dead leaves in the distance as a suede in the third picture against a complex background. The improved YOLOX-s effectively improves such false detections and still accurately detects the target even in the dark night with a complex background.

**Fig. 7:**
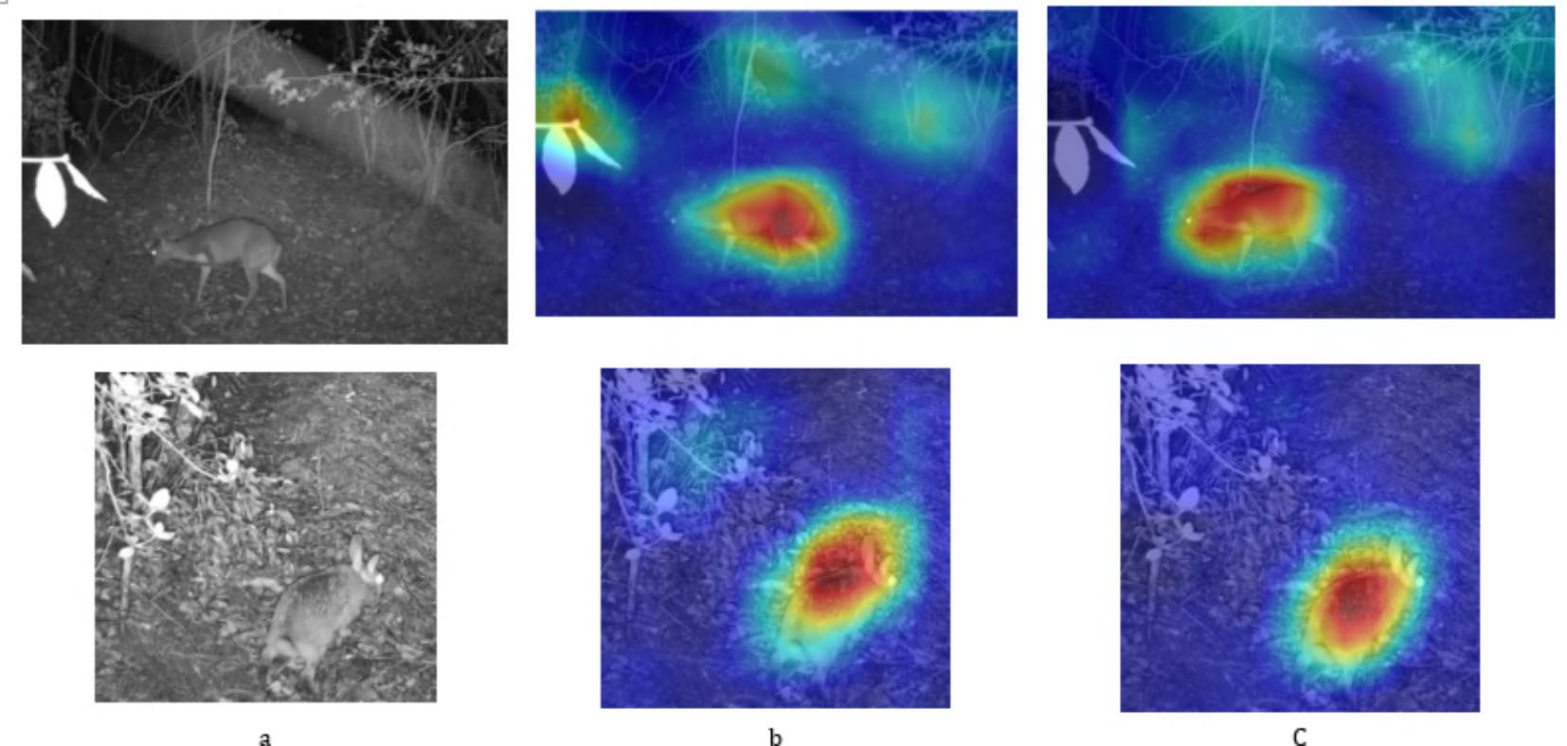
(**a**) The original image of the target at night, (**b**) is the heatmap generated by YOLOX, and (**c**) is the heatmap generated by the algorithm presented in this paper.

As shown in Fig. 7, in the YOLOX-generated heatmap (b), apart from the animal, there are other areas that display elevated temperature values, representing background noise. Conversely, the heatmap (c) produced by the algorithm in this study seems to mitigate background noise more effectively, as evidenced by its cooler background hues, which suggests it may suppress irrelevant thermal signals more efficiently. Both heatmaps accurately delineate the animal’s position, but the one from the current study (c) shows a higher concentration of color intensity at the target site, implying a more precise target localization.

## Conclusion

This paper introduces a wildlife object detection algorithm based on the improved YOLOX-s network. Leveraging the MobileViT attention mechanism, we proposed MobileViT-Pooling to extract features from the three feature layers output by the main network, thereby enhancing the weighting of pertinent information. Additionally, we adopted the Dynamic Head, a dynamic object detection head that unifies both the object detection head and attention. Within scale-aware feature layers, spatially-aware spatial positions, and task-aware output channels, it seamlessly integrates the multi-head self-attention mechanism, significantly enhancing the representation ability of the object detection head. Finally, we improved the IoU loss function by incorporating the Focal concept to balance negative samples. Experimental results demonstrate that the enhanced YOLOX-s network achieved an mAP@0.5 of 87.8%, marking a 7.9% increase, and mAP@0.5:0.95 of 62.0%, representing a 5.3% boost compared to the original algorithm. This signifies a significant improvement in accuracy. However, there are still shortcomings in our research. The dataset collected by our algorithm needs further expansion, and the diversity of included animal species is not comprehensive enough. While our algorithm exhibits higher accuracy in nighttime detection compared to others, the precision of nighttime detection alone still requires further enhancement. We are continuously expanding this dataset by incorporating more animal species and images to facilitate broader and more in-depth research. Additionally, we will consistently integrate transformations to enhance the algorithm’s accuracy in detecting complex scenarios during dark nights.

## Data Availability

The dataset of this paper will not be made public. If there are specific requirements, individuals can contact the corresponding author via email to request access to the dataset.
